# Donor-specific anti-human leukocyte antigen antibodies were associated with primary graft failure after unmanipulated haploidentical blood and marrow transplantation: a prospective study with randomly assigned training and validation sets

**DOI:** 10.1186/s13045-015-0182-9

**Published:** 2015-07-10

**Authors:** Ying-Jun Chang, Xiang-Yu Zhao, Lan-Ping Xu, Xiao-Hui Zhang, Yu Wang, Wei Han, Huan Chen, Feng-Rong Wang, Xiao-Dong Mo, Yuan-Yuan Zhang, Ming-Rui Huo, Xiao-Su Zhao, Kong Y, Kai-Yan Liu, Xiao-Jun Huang

**Affiliations:** Peking University People’s Hospital, Peking University Institute of Hematology, Beijing Key Laboratory of Hematopoietic Stem Cell Transplantation, No. 11 South Street of Xizhimen, Xicheng District, Beijing, 100044 Peoples’ Republic of China; Peking-Tsinghua Center for Life Sciences, Beijing, 100871 China; Collaborative Innovation Center of Hematology, Peking University, Beijing, China

## Abstract

**Background:**

Small studies suggest an association of donor-specific anti-human leukocyte antigen (HLA) antibodies (DSAs) with primary graft failure (GF) following haploidentical stem cell transplantation, but primary graft rejection (GR) was not discriminated from primary poor graft function (PGF). In this study, we aimed to determine the association of DSAs with primary GF, including GR and PGF, in patients who underwent unmanipulated haploidentical blood and marrow transplantation.

**Methods:**

A total of 345 subjects were prospectively recruited and randomly selected as training group (*n* = 173) and validation group (*n* = 172). Patient plasma/serum was screened. For HLA antibody positive samples with a median fluorescent intensity (MFI) >500, DSAs were further tested using a LABScreen Single Antigen Kit (One Lambda).

**Results:**

A total of 342 patients (99.1 %) achieved sustained myeloid engraftment. The median times to neutrophil engraftment and platelet engraftment were 13 days (range, 8–28 days) and 18 days (range, 6–330 days), respectively. The cumulative incidence of primary GF was 6.4 ± 1.3 % and included GR (0.9 ± 0.5 %) and PGF (5.5 ± 1.2 %). Of the 345 cases tested, 39 (11.3 %) were DSA positive. Multivariate models showed that DSAs (MFI ≥ 10,000) were correlated to primary GR (*P* < 0.001) and that DSAs (MFI ≥ 2000) were strongly associated with primary PGF (*P* = 0.005). All patients were classified into three groups for analysis. Group A included cases that were DSA negative and those with a DSA MFI <2000 (*n* = 316), group B included cases with a 2000 ≤ MFI < 10,000 (*n* = 19), and group C included cases with a MFI ≥10,000 (*n* = 10). The DSAs were associated with an increased incidence of the primary GF (3.2 vs. 31.6 vs. 60 %, for groups A, B, and C, respectively, *P* < 0.001), transplant-related mortality (TRM) rate (17.2 vs. 14.7 vs. 33.3 %, for groups A, B, and C, respectively, *P* = 0.022), and inferior overall survival (OS, 77.3 vs. 85.3 vs. 44.4 %, for groups A, B, and C, respectively, *P* = 0.015). The primary GF was independently associated with a higher incidence of TRM (*P* < 0.001), inferior disease-free survival (*P* < 0.001), and OS (*P* < 0.001).

**Conclusions:**

The findings confirmed the effect of DSAs on primary GF, including GR and PGF, and survival. Our results suggest incorporating DSAs in the algorithm for haploidentical donor selection.

## Background

Allogeneic stem cell transplantation (allo-SCT) is a potentially curative treatment for patients with hematologic malignancies [1–7]. However, complications, such as graft failure (GF) and relapse, remain serious problems [4, 5, 8–16]. Primary GF includes graft rejection (GR), which is defined as a failure to engraft neutrophils (absolute neutrophil count (ANC) ≤0.5 × 10^9^/L) by day +28 for three consecutive days and the absence of donor hematopoiesis [14, 17]. It also includes poor graft function (PGF), which is the failure to achieve two or three adequate blood counts (ANC ≤0.5 × 10^9^/L, platelet ≤20 × 10^9^/L, or hemoglobin (Hb) ≤80 g/L) following allo-SCT in the presence of complete donor hematopoiesis [12, 14, 17]. The incidence of primary GF ranges from 2 to 15 % in patients who undergo human leukocyte antigen (HLA)-matched sibling donor transplantation, unrelated donor transplantation (MUDT), or umbilical cord blood transplantation (UCBT) [10, 13, 18–22]. In the past 10 years, HLA-mismatched/haploidentical transplants (haplo-SCTs) have been used more frequently [1–5, 23–25]; as a result, GF has become an increasing problem that contributes to high morbidity and mortality after transplantation. The incidences of GF in patients who underwent CD34-selected [5] and CD3/CD19-depleted haplo-SCTs [4] were 9 and 8 %, respectively. The rate of GF following haplo-SCTs with post-transplant cyclophosphamide was 13 % [8, 25].

Donor-specific antibodies (DSAs) refer to anti-HLA antibodies that specifically correspond to a mismatched antigen of the donor [9, 20–22, 26–28]. The role of DSAs in solid organ transplantation is well established [29]. In allo-SCTs [20, 21, 30], DSAs have been associated with primary GF after either MUDT or UCBT. In haplo-SCT, Ciurea et al. [22] showed that 75 % of pretransplant DSA-positive patients with a median fluorescent intensity (MFI) >1500 failed to engraft, compared with 5 % of DSA-negative patients (*P* = 0.008). In another study, the authors found that three of five patients with high levels of DSA (MFI > 10,000) had GF. [9] Although the association of DSAs with primary GF after haplo-SCT has been observed [9, 22, 31], there are some limitations of previous studies: (1) most studies were retrospective [22, 31], except one [9]; (2) they included small numbers of patients [9, 22, 31]; (3) primary GR was not discriminated from primary PGF [9, 22, 31]; and (4) there were no training and validation groups [9, 22, 31].

In our center, we established an unmanipulated haploidentical blood and marrow transplant (HBMT) protocol that can achieve outcomes comparable with HLA-identical sibling or unrelated donor transplantation. The incidence of primary GR was approximately 1 % in patients undergoing unmanipulated HBMT. [1, 32] However, we found that primary PGF, with an incidence of approximately 4–5 %, was a severe complication with a higher incidence of mortality after unmanipulated HBMT (unpublished data). Therefore, we prospectively investigated the influence of DSAs on primary GF, including GR and PGF, after unmanipulated HBMT in a training group of 173 patients and validated the results in an independent cohort of 172 cases.

## Results

### Patient characteristics

The median age of the patients was 26 years (range, 2–58 years). All patients were treated with a myeloablative conditioning regimen. The median infused total nucleated cell dose (TNC) and CD34^+^ cell dose were 8.34 × 10^8^/kg (range, 1.78–23.69 × 10^8^/kg) and 2.59 × 10^6^/kg (range, 0.39–16.82 × 10^6^/kg), respectively. Other demographics are listed in Table 1. The characteristics of the patients in the training group and validation set were similar.

**Table 1:** Patient and donor characteristics

	All patients	Training group	Validation group	*P* value
Patient number	345	173	172	
Median age (range), years	26 (2–58)	26 (4–58)	26 (2–58)	0.830
Median weight (range), kg	62 (13–113)	61 (18–113)	63 (13–110)	0.388
Male sex, *n* (%)	201 (58.3)	100 (57.8)	101 (58.7)	0.863
Diagnosis, *n* (%)				0.142
AML	137 (39.7)	69 (39.9)	68 (39.5)	
ALL	116 (33.6)	49 (28.3)	67 (39.0)	
CML	19 (5.5)	10 (5.8)	9 (5.2)	
MDS	38 (11.0)	24 (13.9)	14 (8.1)	
Others	35 (10.1)	21 (12.1)	14 (8.1)	
Disease status, SR/HR (*n* (%))	265 (76.8)/80 (23.2)	135 (78.0)/38 (22.0)	135 (78.0)/38 (22.0)	0.589
Conditioning regimen				
MA	345 (100 %)	173 (100 %)	172 (100 %)	
No of HLA-A, B, DR mismatched				0.302
0	2 (0.6 %)	1 (0.6 %)	1 (0.6 %)	
1	18 (5.2 %)	13 (7.5 %)	5 (2.9 %)	
2	74 (21.4 %)	34 (19.7)	40 (23.3 %)	
3	249 (72.2 %)	124 (71.1 %)	125 (72.2 %)	
Donor-recipient sex match, *n* (%)				0.609
Male-male	132 (38.3)	63 (36.4)	69 (40.1)	
Male-female	89 (25.8)	42 (24.3)	47 (27.3)	
Female-male	71 (20.6)	38 (22.0)	33 (19.2)	
Female-female	53 (15.4)	30 (17.3)	23 (13.4)	
Donor-recipient relationship, *n* (%)				0.419
Father-child	134 (38.8)	64 (37.0)	70 (40.7)	
Mother-child	45 (13.0)	28 (16.2)	17 (9.9)	
Sibling-sibling	103 (29.9)	53 (30.6)	50 (29.1)	
Child-parent	53 (15.4)	25 (14.5)	28 (16.3)	
Other	10 (2.9)	3 (1.7)	7 (4.1)	
ABO matched, *n* (%)				0.345
Matched	196 (56.8)	102 (59.2)	94 (54.7)	
Major mismatched	64 (18.6)	34 (19.7)	30 (17.4)	
Minor mismatched	18 (5.2)	10 (5.8)	8 (4.7)	
Bidirect mismatched	67 (19.4)	27 (15.6)	40 (23.3)	
Cell compositions in allografts				
Infused nuclear cells 10^8^/kg	8.34 (1.78–23.69)	8.32 (1.78–23.59)	8.40 (2.12–23.69)	0.321
Infused CD34^+^ cells 10^6^/kg	2.59 (0.39–16.82)	2.71 (0.39–14.47)	2.49 (0.58–16.82)	0.840
Infused lymphocytes 10^8^/kg	2.95 (0.16–9.49)	2.88 (0.16–9.49)	3.06 (0.68–7.34)	0.107
Infused CD3^+^ cells 10^8^/kg	2.00 (0.10–5.93)	2.00 (0.10–5.93)	1.99 (0.20–5.36)	0.575
Infused CD4^+^ cells 10^8^/kg	1.10 (0.10–3.94)	1.09 (0.10–3.33)	1.13 (0.19–3.94)	0.371
Infused CD8^+^ cells 10^8^/kg	0.70 (0.05–2.47)	0.69 (0.05–2.47)	0.72 (0.14–2.43)	0.452
Infused CD14^+^ cells 10^8^/kg	1.49 (0.05–4.90)	1.50 (0.33–4.90)	1.48 (0.05–6.13)	0.769

*Abbreviations*: *AML* acute myeloid leukemia, *ALL* acute lymphoblastic leukemia, *CML* chronic myeloid leukemia, *MDS* myelodysplastic syndrome, *HLA* human leukocyte antigen, *BM* bone marrow

### Transplant outcomes

A total of 342 patients (99.1 %) achieved sustained myeloid engraftment. The median times to neutrophil engraftment and platelet engraftment were 13 days (range, 8–28 days) and 18 days (range, 6–330 days), respectively. The cumulative incidence of primary GF was 6.4 ± 1.3 % and included GR (*n* = 3, 0.9 ± 0.5 %) and PGF (*n* = 19, 5.5 ± 1.2 %). At 100 days after transplant, the cumulative incidence of grade 2 to 4 acute graft-versus-host disease (GVHD) was 42.7 ± 3.1 %. After a median follow-up of 384 days (range, 25–784 days), the cumulative incidence of chronic GVHD was 43.3 ± 3.1 %. The 2-year probabilities of relapse, transplant-related mortality (TRM), disease-free survival (DFS), and overall survival (OS) were 8.8 ± 1.8 %, 18.4 ± 2.8 %, 75.1 ± 2.9 %, and 76.2 ± 3.0 %, respectively.

### Anti-HLA antibodies and DSAs

Of the 345 cases tested, 87 (25.2 %) were anti-HLA antibody positive, including 44 males and 43 females. Of the positive cases, 39 (11.3 %) were DSA positive. Among the 39 cases, 31 had antibodies against HLA class I antigens, 15 had antibodies against HLA class II, and 7 against classes I and II. The MFI was 4726 (range, 504–19,948). Among 144 female cases, the patients with a pregnancy history had a higher anti-HLA antibody positive rate (100 vs. 17.2 %, *P* < 0.001) and a higher DSA positive rate (59.1 vs. 8.2 %, *P* < 0.001) than those without.

### Association of DSAs on primary GR after transplantation

In this study, we defined a MFI ≥ 10,000 of DSAs as a cutoff value for primary GR using receiver operating characteristic curves in all the 345 patients who underwent unmanipulated HBMT due to the low incidence of primary GR [1, 2, 32]. The incidence of primary GR with a MFI ≥10,000 was higher than those with a MFI <10,000 (20 vs. 0.3 %, *P* = 0.002) in all the 345 patients. The numbers of patients with primary GR in training and validation sets were two cases and one case, respectively. The higher incidences of primary GR in patients with a MFI ≥ 10,000 than those with a MFI < 10,000 were also observed in the training group (16.7 vs. 0.6 %, *P* = 0.041) and the validation group (25.0 vs. 0 %, *P* = 0.023). Univariate analysis showed that factors, including age (*P* = 0.002), disease status (*P* = 0.018), donor-recipient relationship (*P* = 0.057), anti-HLA antibodies (*P* < 0.001), and DSAs (*P* < 0.001), were correlated with primary GR after unmanipulated HBMT. Multivariate analysis demonstrated that the presence of DSAs (MFI ≥ 10,000) was associated with primary GR (hazard ratio (HR) 71.556, 95 % confidence interval (CI) 6.488–789.129; *P* < 0.001). The onset of primary GR was associated with increased TRM (HR 18.893, 95 % CI 5.538–64.452; *P* < 0.001), inferior DFS (HR 9.883, 95 % CI 3.005–32.502; *P* < 0.001), and OS (HR 11.747, 95 % CI 3.546–38.916; *P* < 0.001).

### Association of DSAs with primary PGF after unmanipulated HBMT

We further investigated the effects of DSAs on primary PGF after unmanipulated HBMT. In the training set (*n* = 173), a cutoff value of a DSA MFI ≥2000 was identified to predict the onset of primary PGF. The patients with a MFI ≥2000 experienced a significantly higher incidence of primary PGF than those with a MFI <2000 [27.3 % (3/11) vs. 1.9 % (3/162), *P* = 0.003]. Multivariate models showed that the presence of DSAs was strongly associated with primary PGF (HR 10.575, 95 % CI 2.029–55.117; *P* = 0.005). The same threshold of DSAs was applied in the independent validation set of the 172 patients. In the validation group, the incidence of primary PGF was higher in patient with a MFI ≥2000 compared with those with a MFI <2000 [33.3 % (6/18) vs. 4.5 % (7/154), *P* = 0.001]. Multivariate analysis further confirmed the presence of DSAs was independently associated with the onset of primary PGF (HR 3.949, 95 % CI 1.501–10.389; *P* = 0.005) after unmanipulated HBMT. After identifying primary GR as a competing risk for primary PGF, the correlation of DSAs with primary PGF was also demonstrated in the training group and the validation group (data not shown).

In the training set, multivariate analysis showed that the onset of primary PGF was independently associated with a higher incidence of TRM (HR 7.114, 95 % CI 2.054–24.639; *P* = 0.002), inferior DFS (HR 3.356, 95 % CI 1.047–10.759; *P* = 0.042), and OS (HR 3.687, 95 % CI 1.129–12.039; *P* = 0.031). These independent associations of primary PGF with a higher incidence of TRM (HR 5.031, 95 % CI 1.993–12.704; *P* = 0.001), inferior DFS (HR 3.011, 95 % CI: 1.247–7.617; *P* = 0.014), and OS (HR 3.530, 95 % CI 1.445–8.626; *P* = 0.006) were confirmed in the validation group.

### Effects of DSA on primary graft failure and transplant outcomes

**Fig. 1:**
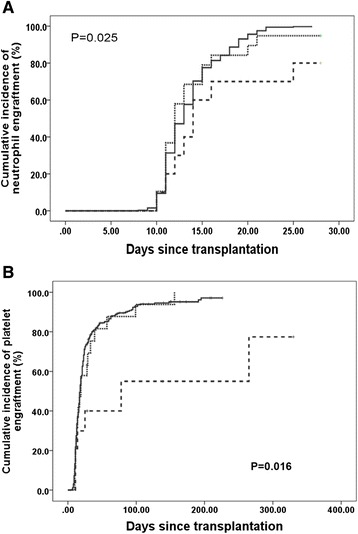
Pretransplant DSA and cumulative incidence of neutrophil (**a**) and platelet (**b**) engraftment. All patients were classified into three groups, group A includes cases with DSA negative and those with a DSA MFI <2000 (*n* = 316, *solid line*), group B includes cases with 2000 ≤ MFI < 10,000 (*n* = 19, *dotted line*), and group C includes those with a MFI ≥ 10,000 (*n* = 10, *dashed line*)

After separately analyzing the association of DSAs with either primary GR or primary PGF, we further investigated the association of DSAs with primary GF, including both GR and PGF, in all the 345 patients. These patients were classified into three groups, group A included cases that were DSA negative or had a DSA MFI <2000 (*n* = 316), group B included cases with a 2000 ≤ MFI < 10,000 (*n* = 19), and group C included those with a MFI ≥10,000 (*n* = 10). The cumulative incidence of neutrophil engraftment of patients in group A was 100 %, which was significantly higher than the cumulative incidence of group C (80.0 ± 12.6 %, *P* = 0.005) and comparable with group B (94.7 ± 5.1 %, *P* = 0.169) (Fig. 1a). The cumulative incidence of platelet engraftment of patients in group A was 97.1 ± 1.30 %, which was significantly higher than the incidences of group B (93.9 ± 5.9 %, *P* = 0.030) and group C (77.5 ± 18.1 %, *P* = 0.004) (Fig. 1b). Multivariate analysis showed that the presence of DSAs was strongly associated with platelet engraftment and primary graft failure, but not neutrophil engraftment (Table 2).

**Table 2:** Multivariate analysis of factors associated with transplant outcomes

	HR	95 % CI	*P* value
Primary graft failure			
DSA			
MFI ≥ 10,000	1		
2000 ≤ MFI < 10,000	0.940	0.284–3.177	*0.919*
MFI < 2000	0.187	0.048–0.730	*0.016*
OS			
Disease status	2.839	1.702–4.736	0.000
Primary graft failure			
GR	1		
PGF	0.271	0.074–1.000	0.050
No primary graft failure	0.068	0.020–0.229	0.000
DFS			
Disease status	3.593	2.212–5.836	0.000
PGF	3.125	1.564–6.244	0.000
GR	1		
PGF	0.284	0.077–1.044	0.058
No primary graft failure	0.084	0.025–0.279	0.000
Relapse			
Disease status	9.906	4.099–23.940	0.000
TRM			
PGF			
GR	1		
PGF	0.209	0.056–0.790	0.021
No primary graft failure	0.031	0.0009–0.107	0.000
ANC			
CD34	1.370	1.106–1.697	0.004
PLT			
CD34	1.483	1.187–1.852	*0.001*
DSA			
MFI ≥ 10,000	1		
2000 ≤ MFI < 10,000	3.074	1.137–8.311	0.027
MFI < 2000	3.301	1.358–8.022	0.008

*Abbreviations*: *HR* hazard ratio, *CI* confidence interval, *DSAs* donor-specific antibodies, *MFI* median fluorescence intensity, *OS* overall survival, *GR* graft rejection, *PGF* poor graft function, *DFS* disease-free survival, *TRM* transplant-related mortality, *ANC* absolute neutrophil count, *PLT* platelet

**Fig. 2:**
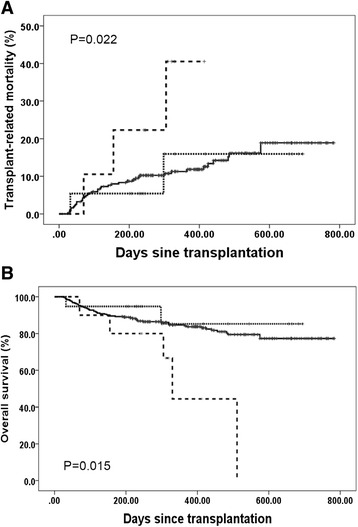
Transplant-related mortality (**a**) and overall survival (**b**). All patients were classified into three groups, group A includes cases with DSA negative and those with a DSA MFI <2000 (*n* = 316, *solid line*), group B includes cases with 2000 ≤ MFI < 10,000 (*n* = 19, *dotted line*), and group C includes those with a MFI ≥10,000 (*n* = 10, *dashed line*)

**Fig. 3:**
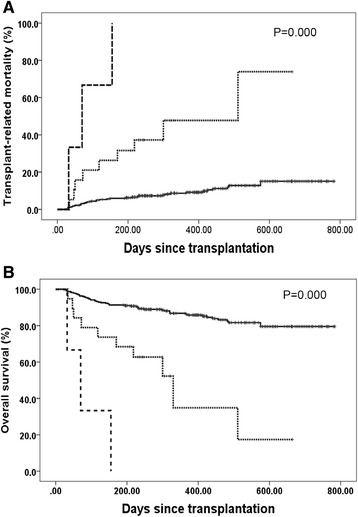
Transplant-related mortality (**a**) and overall survival (**b**). The *solid line*, *dotted line*, and *dashed line* represents patients without primary graft failure, with graft rejection, and with poor graft function, respectively

The incidences of primary GF, including GR and PGF, in groups A, B, and C were 3.2 % (10/316), 31.6 % (6/19), and 60 % (6/10), respectively (*P* < 0.001). The cumulative incidences of the TRM rate were 17.2 %, 14.7 %, and 33.3 %, for patients in groups A, B, and C, respectively (Fig. 2a, *P* = 0.022). The overall survival rates were 77.3, 85.3, and 44.4 % for patients in groups A, B, and C, respectively (Fig. 2b, *P* = 0.015). Multivariate analysis showed that the presence of DSAs was strongly associated with primary GF (Table 2). The onset of primary GF was also independently associated with a higher incidence of TRM and inferior DFS and OS (Table 2 and Fig. 3a, b). As shown in Table 3, the causes of death in patients with primary GF were mainly infections and hemorrhage, which occurred significantly more often than those without GF (*P* < 0.001). There were no effects of DSAs on GVHD and relapse (Table 2).

**Table 3:** Causes of death for patients who underwent unmanipulated HBMT

Causes of death^a^	All patients (*n* = 345)	Patients with primary GF (*n* = 22)	Patients without primary GF (*n* = 323)
Relapse	18 (5.2 %)	1 (4.5 %)	17 (5.3 %)
Infections	27 (7.8 %)	8 (36.4 %)	19 (5.9 %)
Hemorrhage	7 (2.0 %)	3 (13.6 %)	4 (1.2 %)
GVHD	3 (0.9 %)	0 (0 %)	3 (0.9 %)
Others	8 (2.4 %)	1 (4.5 %)	7 (2.1 %)

*Abbreviations*: *HBMT* haploidentical blood and marrow transplantation, *GF* graft failure, *GVHD* graft-versus-host disease

^a^Indicate the number (%) of patients

## Discussion

We confirmed the association of DSAs with primary GF, as previously reported [9, 22], in this prospective study with randomly assigned training and validation sets. This finding along with the results reported by others [9, 10, 15, 16, 20–22, 28, 30] suggests that the presence of DSAs may contribute to the pathophysiology of GF not only in MUDT and UCBT but also in haplo-SCT with T cell depletion or T cell replete. Most importantly, for the first time, we found a correlation between the presence of DSAs and primary PGF, indicating that DSAs may be involved in the pathogenesis of this complication. The finding that primary GF, including both GR and PGF, can result in inferior OS provides evidence that the presence of DSAs must be considered when choosing a haploidentical donor and should be incorporated in the donor selection algorithm [2, 33].

Our previous reports of a low incidence of primary GR [32, 34] and the association of DSAs with primary GR led us to this investigation of the effects of DSAs on primary PGF in patients receiving our haploidentical transplant protocol. [1, 2, 32, 34] Importantly, we identified for the first time that a MFI ≥2000 was the DSA threshold for primary PGF after haplo-SCT. Our results demonstrated that the presence of DSAs was strongly associated with the onset of primary PGF, in both the training and validation sets. Moreover, we found that primary PGF was an independent variable, which led to inferior survival. Therefore, except for CD34(+)-selected stem cell boost and other methods [13, 17], targeting DSAs may provide a novel method to treat PGF, although the DSA MFI threshold for primary PGF needs to be confirmed in other haploidentical transplant modalities.

The definition of a threshold for DSAs, according to MFI, is a premise for analyzing the association of DSAs with primary GF. In CBTs, Takanashi et al. [30] considered a MFI >1000 to be DSA positive. In a case-control study conducted by Ciurea et al. [20], a MFI ≥500 was considered positive. In haplo-SCT, MFI values >1500 or 5000 were defined as DSA positive by Ciurea et al. [22] and Yoshihara et al. [9], respectively. In our study, we identify a MFI ≥10,000 and MFI ≥2000 as the cutoff values for primary GR and primary PGF, respectively. The differences in the reported thresholds of DSAs between other studies [9, 20–22] and this report may be related to different transplant protocols and different methods for DSA detection [9, 10, 15, 16, 20–22, 28, 30], although these studies demonstrated that the antibody titer is important for the effects of DSAs on primary GF. In addition, we observed that high and low antibody titers of DSAs led to GR and PGF, respectively. Both GR and PGF contributed to inferior survival, although the survival was reduced in GR compared with PGF (Fig. 3). Therefore, our results suggest that high and low MFIs of DSAs should be dealt with differently.

After investigating the association of DSAs with GR and PGF, respectively, we further investigated this association of DSAs with primary GF by classifying all the 345 patients into three groups according to the cutoff value of the DSA MFI. We found that patients with a DSA MFI ≥10,000 experienced a significantly lower cumulative incidence of platelet engraftment, but not neutrophil engraftment, after multivariate analysis. This finding is in agreement with a previous study [9]. The result of the lack of an effect of DSAs on neutrophil engraftment may be related to the routine use of G-CSF in our transplant protocol. [1, 32] Moreover, the effect of DSAs on primary PGF was also demonstrated in the overall cohort. As demonstrated by Cutler et al. [21] in CBTs, we showed that pretransplant DSAs were associated with a higher TRM rate and inferior survival, although a multicenter study with a larger sample of cases is needed to confirm these effects in multivariate analysis. Our study results support the logical theory that the presence of DSAs results in primary GF and may contribute to inferior survival.

Previous studies demonstrated that DSAs may kill donor cells through antibody-dependent cell-mediated cytotoxicity (ADCC) [26], indicating that an immune-mediated mechanism may contribute to the pathogenesis of primary GF. In the present study, the findings that a high MFI of DSAs led to GR and a low MFI resulted in PGF suggest that not only the onset GR but also PGF may involve immune-mediated mechanisms. In renal transplantation, DSAs may result in allograft injury though endothelial cell apoptosis [35, 36]. In systemic sclerosis, the ADCC effect via the Fas pathway can lead to bone marrow (BM) endothelial cell apoptosis [37]. Based on previous reports [35, 36] and the results of this study, it is conceivable that higher titers of DSAs directed against antigens expressed by all full donor cells may lead to necrosis, resulting in primary GR. While, lower titers of DSAs directed against antigens of donor cells may cause apoptosis and the onset of primary PGF. [14, 15] Our study suggests that abnormalities in the BM microenvironment, especially endothelial progenitor cells (EPCs), may cause PGF. [14] Therefore, the effects of DSAs on the BM microenvironment, especially EPCs, during the development of primary GF should be further investigated.

## Conclusions

The findings of this study confirmed the effects of DSAs on primary GR. Impressively, we, for the first time, demonstrated that the presence of DSAs might contribute to the pathogenesis of primary PGF after unmanipulated HBMT. Due to the involvement of DSAs in primary GF, including GR and PGF, and inferior survival, the proportions of DSAs can be used in haploidentical transplant settings to decide who is the best donor [2, 33].

## Methods

### Study cohort

Patients who underwent unmanipulated HBMT were eligible for this study and prospectively enrolled. All cases were treated according to our institutional transplant protocol, as previously described in detail [1, 32, 34]. A total of 345 subjects were recruited between May 2012 and March 2014. These cases were randomly selected as part of the training group (*n* = 173) or validation group (*n* = 172). The protocol was approved by the Institutional Review Board of Peking University and signed informed consent was obtained from all the subjects. This study was conducted in accordance with the Declaration of Helsinki.

### Transplant protocol

Institutional protocols for unmanipulated HBMT have been previously described [32, 34]. In brief, the conditioning therapy was consisted of cytarabine (4 g/m^2^/day, on days −10 to −9), busulfan (3.2 mg/kg/day, intravenously on days −8 to −6), cyclophosphamide (1.8 g/m^2^/day, on days −5 to −4), 1-(2-chloroethyl)-3-(4-methylcyclohexyl)-1-nitrosourea (Me-CCNU, 250 mg/m^2^, once on day −3), and ATG (2.5 mg/kg/ day, rabbit; Sang Stat, Lyon, France, on days −5 to −2) [38]. All transplant recipients received mixture allografts of G-CSF-mobilized bone marrow and peripheral blood stem cell harvests. Cyclosporine A, mycophenolate mofetil, and short-term methotrexate were used for prophylaxis of GVHD [39]. Cytomegalovirus (CMV) and Epstein-Barr virus (EBV) monitoring and prevention and donor lymphocyte infusion were performed according to previous studies [34, 40, 41].

### Methodology to detect anti-HLA antibodies

Patient plasma/serum was screened for class I (i.e., HLA-A,-B,-C) and class II (i.e., HLA-DR) HLA antibodies with a LABScreen Mixed Kit (One Lambda, Canoga Park, CA, USA). The samples (7 uL) were incubated with mixed HLA class I- and class II-coated microspheres for 30 min in the dark under gentle agitation. The specimens were then washed before being incubated with anti-human immunoglobulin G-conjugated fluorescein isothiocyanate as described above for the first incubation. Next, the samples were analyzed with a Luminex 200 flow analyzer (Luminex, Austin, TX, USA), and the data were analyzed with the HLA Fusion 3.2 software (One Lambda). The MFI of anti-HLA antibodies was obtained from the output file generated by the flow analyzer and adjusted for the background signal using the formula: sample beads − negative control beads. The samples with a MFI >500 were further tested for the specificity of the antibody, using a LABScreen Single Antigen Kit (One Lambda). The MFI was adjusted for the background signal using the formula described above. The patients and donors underwent HLA allele typing of at least the A, B, and DRB1 loci routinely.

### Definitions and evaluation

Neutrophil engraftment was defined as achieving an ANC of 0.5 × 10^9^/L or greater for three consecutive days, and platelet recovery was defined as achieving a platelet count of 20 × 10^9^/L or greater, without platelet transfusions, for seven days. A Hb level of at least 80 g/L without transfusion support is the accepted threshold for red cell engraftment [12]. Full donor chimerism was defined as ≥95 % leukocytes of donor origin in peripheral blood or marrow samples, measured according to our previous report. [14] Mixed chimerism was defined as more than 5 % but less than 95 % leukocytes of donor origin.

Primary GF included GR and PGF. As described in the introduction, GR is the failure to engraft neutrophils (ANC ≤0.5 × 10^9^/L) by day +28 for three consecutive days and the absence of donor hematopoiesis. Because delayed red cell engraftment may happen for many months post-transplant and is more difficult to evaluate in an unarguable manner, in the present study, primary PGF was defined as the presence of three cytopenic counts (ANC ≤0.5 × 10^9^/L, platelet ≤20 × 10^9^/L, or hemoglobin (Hb) ≤80 g/L) beyond day +28 with a transfusion requirement associated with hypoplastic-aplastic BM, in the presence of complete donor chimerism. The patients with evidence of severe GVHD or hematologic relapse were excluded [14].

The diagnosis and grading of acute and chronic GVHD was assigned by the transplant center using standard criteria [42, 43]. Transplant-related mortality (TRM), relapse, DFS, and overall survival (OS) was defined according to our previous studies [2, 32, 34].

### Statistical analysis

The patient baseline characteristics were reported descriptively. The Fisher exact test or Wilcoxon rank sum test was used for two-group comparisons. Death without engraftment was considered a competing risk for engraftment, primary GR, and primary PGF, while primary GR was considered a competing risk for primary PGF. The surviving patients were censored at their date of last known follow-up. The log-rank test was used for comparisons of Kaplan-Meier curves, and a Gray test was used for comparisons of cumulative incidence curves. Potential prognostic factors for OS, DFS, relapse, TRM, and engraftment were examined in proportional hazards models. To explore whether the DSA intensity, measured as MFI, predicted primary GF, including GR and PGF, an analysis of receiver operator characteristics was performed. Cox regression models were developed to test the impact of variables on primary GF. Unless otherwise specified, *P* values are based on two-sided hypothesis tests. Alpha was set at 0.05. We used SPSS 16.0 (Mathsoft, Seattle, WA, USA) for most analyses.
